# Improving 3D Genome Reconstructions Using Orthologous and Functional Constraints

**DOI:** 10.1371/journal.pcbi.1004298

**Published:** 2015-05-22

**Authors:** Alon Diament, Tamir Tuller

**Affiliations:** 1 Dept. of Biomedical Engineering, Tel Aviv University, Tel Aviv, Israel; 2 The Sagol School of Neuroscience, Tel Aviv University, Tel Aviv, Israel; CNAG - Centre Nacional d’Anàlisi Genòmica and CRG - Centre de Regulació Genòmica, SPAIN

## Abstract

The study of the 3D architecture of chromosomes has been advancing rapidly in recent years. While a number of methods for 3D reconstruction of genomic models based on Hi-C data were proposed, most of the analyses in the field have been performed on different 3D representation forms (such as graphs). Here, we reproduce most of the previous results on the 3D genomic organization of the eukaryote *Saccharomyces cerevisiae* using analysis of 3D reconstructions. We show that many of these results can be reproduced in sparse reconstructions, generated from a small fraction of the experimental data (5% of the data), and study the properties of such models. Finally, we propose for the first time a novel approach for improving the accuracy of 3D reconstructions by introducing additional predicted physical interactions to the model, based on orthologous interactions in an evolutionary-related organism and based on predicted functional interactions between genes. We demonstrate that this approach indeed leads to the reconstruction of improved models.

## Author Summary

Understanding the importance of genome architecture, the arrangement of genes within the genome and how this organization evolved has been intensively studied in recent years. Despite rapid progress in the field, accurate 3D modeling of genome organization remains a challenge. While a number of methods for 3D reconstruction of genomic models based on genome-wide experimental data were proposed, most of the analyses in the field have been performed on different 3D representation forms (such as graphs). Here, we reproduce most of the previous results on the 3D genome organization of the eukaryote *Saccharomyces cerevisiae* using analysis of 3D reconstructions. We show that many of these results can be reproduced in sparse reconstructions, generated from a small fraction of the experimental data (5% of the data), and study the properties of such models. Finally, we propose for the first time a novel approach for improving the accuracy of 3D reconstructions by introducing additional predicted physical interactions to the model, based on orthologous interactions in a different organism and based on predicted functional interactions between genes. Our proposed approach can facilitate future studies of 3D genome organization via improved models.

## Introduction

Understanding the importance of genome architecture, the arrangement of genes within the genome, and how this organization evolved has been intensively studied in recent years [1–4]. It has become evident that the genomic architecture and thus the three dimensional organization of genes in the genome is far from random. A recent experimental approach for studying the three-dimensional architecture of genomes, Chromosome Conformation Capture (3C) [5]—and its high-throughput variants (such as Hi-C [6])—has enabled far more accurate characterization of genomic spatial organization.

Different methods have been applied and developed in recent years for the analysis of Hi-C data. Contact frequencies—*i*.*e*., the number of times each pair of genomic loci was observed in proximity—are the raw variables measured in Hi-C experiments, and provide a way to assess the co-localization of sets of loci in the nucleus. Indeed, many of the published results in the field are based on direct analysis of contact frequencies [6–10]. Hi-C data was also studied in conjunction with polymer simulations in order to develop models that may explain the observed contact frequency maps [6,11–13]. For example, a study in *Saccharomyces cerevisiae* has suggested that many earlier results—including features of the contact maps, the co-localization of early firing replication origins and genomic location of tRNA genes—can be explained by random configurations of chromosomes that are tethered to a number of sites in the nucleus [11]. Such random models offer insights into the possible mechanisms that give rise to the complex genomic architecture.

There have been a number of attempts to interpret Hi-C data by generating non-random 3D reconstructions based on distance constraints obtained from contact frequency maps [8,9,14,15]. Such models may have several benefits: reducing noise and biases in the data by seeking consistent solutions for the entire genome; increasing the resolution of the model by generating a continuous solution from discrete samples; enabling a clear interpretation of distances in consistent units (compared with contact frequencies); enabling accurate analysis of loci dispersion as well as co-localization (contact enrichment analysis being limited to the latter); enabling the utilization of existing algorithms for 3D model analysis, such as structural comparison between models; and providing an integrative view of genomic architecture, given the experimental data as well as known physical constraints [8]. Thus 3D reconstructions are a promising way of studying the genomic architecture; nevertheless, most of the previous results have yet to be studied in 3D models, with a few exceptions [14,16–18].

Here we carry out a detailed analysis of the properties of populations of 3D reconstructions of the *S*. *cerevisiae* genome, showing that previous results can be reliably reproduced in 3D models. We quantify the redundancy in information in the previously generated *S*. *cerevisiae* Hi-C map [8], showing that the hallmarks of 3D genomic architecture emerge from a sparse set of distance constraints. Finally, we propose novel ways of improving 3D reconstructions methods by adding orthologous spatial interactions from the fission yeast *S*. *pombe* as well as predicted spatial interactions.

## Results

**Fig 1:**
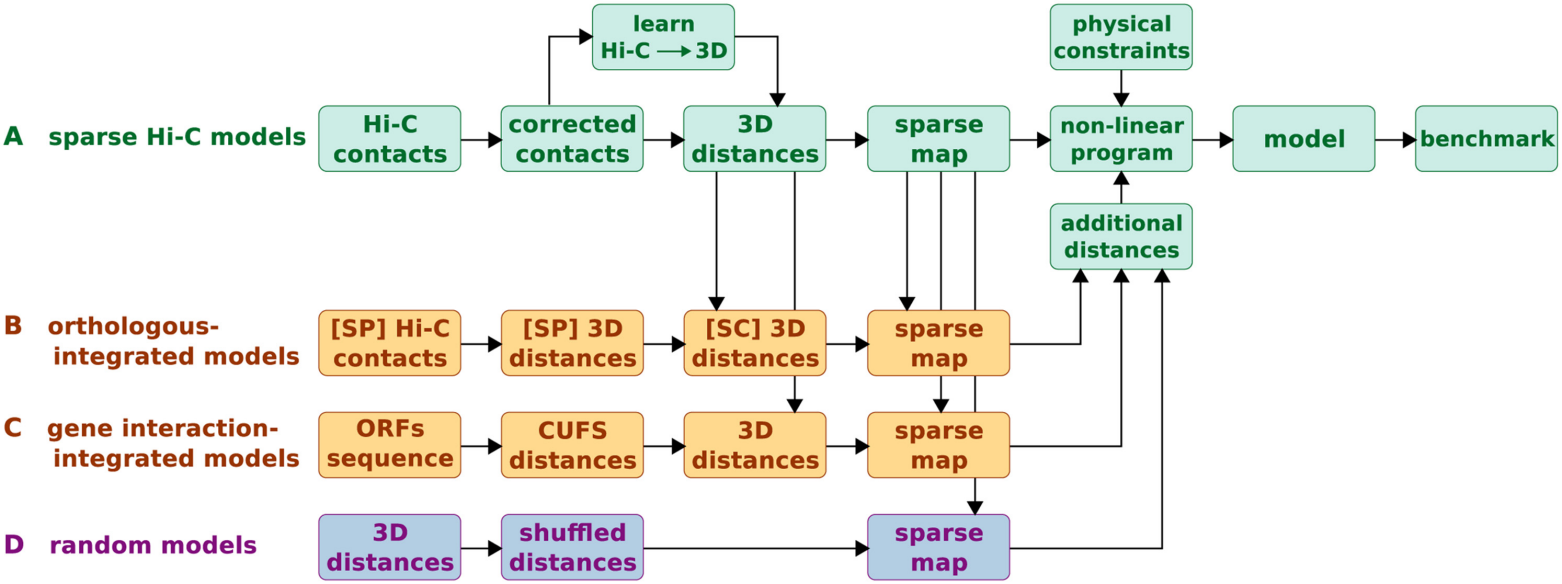
3D reconstruction schemes. The figure depicts the 4 reconstruction schemes employed in the study. **(A)** Sparse Hi-C models were generated by converting Hi-C contact maps to spatial nanometric distances and uniformly sampling from this map according to the desired sparseness. The non-linear program solved by Duan *et al*. [8] was utilized to generate the 3D model. Finally, the quality of the resultant models was assessed in a series of tests based on previously published results on yeast genomic organization. **(B)** Orthologous-integrated models were generated by converting the *S*. *pombe* (SP) Hi-C contact map to spatial nanometric distances, projecting it on the *S*. *cerevisiae* (SC) genome through orthologous genes and normalizing it according to SC distances. Integration into the Hi-C based map of distances was done by sampling non-overlapping (unknown) distances. **(C)** Gene interaction-integrated models were generated by utilizing the codon usage frequency similarity (CUFS) to predict the functional distance between genes. Distances were normalized according to *S*. *cerevisiae* distances, and non-overlapping distances were sampled and integrated into the model. **(D)** Random models were generated by shuffling the coordinates of the Hi-C distance map and integrating them into the model in the same manner.

We adopted the 3D model reconstruction approach proposed by Duan *et al*. [8], a method for generating a consensus model from Hi-C data using a non-linear optimization problem: Chromosomes are composed of beads on a string and their most probable conformation is sought via an objective function, which is based on the observed contact frequencies between DNA segments and minimized under a set of physical constraints (details in Materials and Methods). This approach has been successfully applied in a number of studies [8,9,17–19]. Other approaches for reconstruction have been applied to this problem [14,15,20–26], with comparable results [21,23]. However, some of these approaches have limitations, such as reconstructing each chromosome independently from others [15,20,24,26], dispersing (rather than ignoring) DNA regions with missing data [24,26] or specifying no physical constraints on the chromatin fiber [14,20–24]. Nevertheless, the methods proposed here are not limited to any particular approach. We utilized Duan's reconstruction protocol to generate and study a number of types of 3D models of the *S*. *cerevisiae* genome (Fig 1): First, we generated models of the genome based on varying portions of the Hi-C data (Fig 1A); second, we generated improved models of the genome by integrating additional Hi-C measurements from *S*. *pombe* (Fig 1B); third, we generated improved models of the genome by integrating the predicted functional distance of genes according to the codon usage frequency similarity (CUFS), *i*.*e*. the similarity in the codon composition of genes [16] (Fig 1C); finally, we confirmed that the observed improvement is indeed due to the additional information introduced to the model by comparing it with perturbed models containing integrated random interactions (Fig 1D). A gallery of examples of reconstructed models appears in S1 Fig.

### Previously identified architectural features can be reproduced using sparse 3D reconstructions

**Fig 2:**
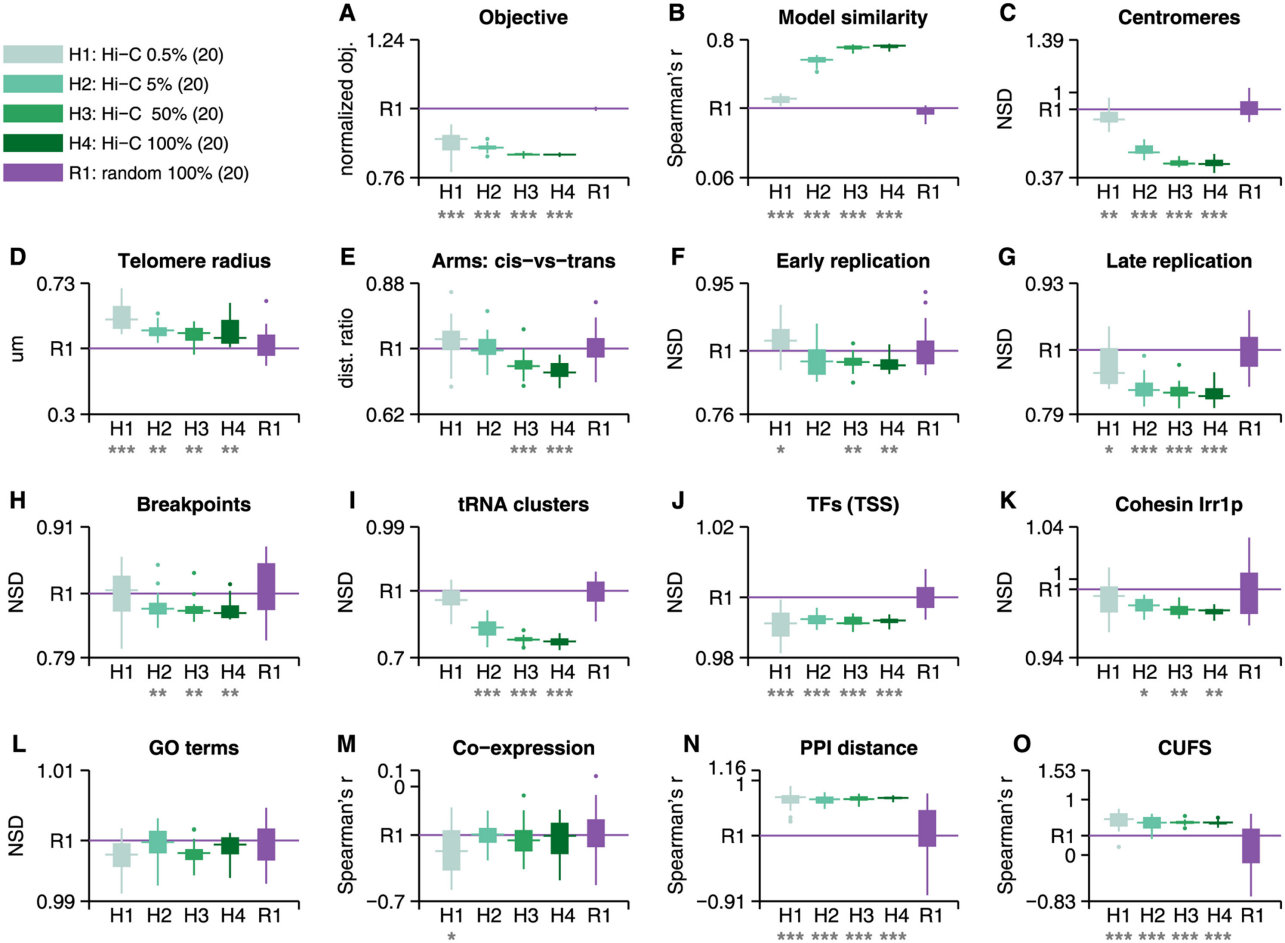
Sparse 3D reconstructions. The figure contains boxplots of the benchmark test results for each of the 5 model categories (H1-4, R1). 20 models were generated from each type. Random models were generated by shuffling the coordinates of the original *S*. *cerevisiae* Hi-C distance map. Results that are distributed significantly above or below the random reference models (their median marked with a line) according to Wilcoxon rank-sum (one-tail), are denoted with a star or more with respect to their significance level (one star for p<0.05, two for p<0.01, three for p<0.001). We observe that on most tests all Hi-C model types obtain similar results, with some tests showing a gradual increase in signal gain with the number of Hi-C interactions. **(A)** Optimization objective function of the reconstructed solution, normalized with respect to random models with similar properties. **(B)** Average Spearman’s correlation between the pairwise distances in each model (9.1x10^5^ points) with the other reconstructions generated in its category. **(C)** Centromere co-localization, measured in normalized set distance (NSD), expected to be lower/greater than 1 for co-localized/dispersed sets, respectively. **(D)** Telomere radius from the center of the nucleus. **(E)** Ratio of the average *cis* (intra-chromosomal) distances between chromosome arms and *trans* (inter-chromosomal) distances. **(F)-(L)** Co-localization results for various sets of functional loci. Where the set comprises of several co-localized subsets (such as each GO term, tRNA clusters 1 and 2, etc.), the result presented is the mean of the sets’ mean distance. **(M)** Spearman's correlation between pairwise distances of genes and their coefficient of correlation of expression (*n* = 2,000 bins). **(N)** Spearman's correlation between pairwise distances of genes and their distances on a protein-protein interaction (PPI) graph (*n* = 2,000 bins). **(O)** Spearman's correlation between pairwise distances of genes and their distances according to the codon usage frequency similarity (CUFS) (*n* = 2,000 bins).

The information in genome-wide Hi-C measurements is expected to be, at least to some degree, redundant [11]. Even for a small set of interactions between DNA loci, physical constraints are expected to considerably reduce the number of probable observed conformations. We studied this effect through sparse Hi-C models, generated by uniformly sampling from the bias-corrected Hi-C map to produce 0.5%, 5% and 50% maps. 20 models were generated in each category (Fig 1A; details in Materials and Methods). We note that sparse reconstructions have been previously generated, based however on highly significant contacts from an FDR-corrected Hi-C map [8,14,19], while here we systematically study a uniform decrease in the amount of data in the map. We then tested whether the models were able to reproduce previously published results that were originally based on analyses of the same *S*. *cerevisiae* Hi-C map (Fig 2). A recent attempt to reproduce some of these results in 3D reconstructions has failed [17], thus we study for the first time 3D genomic models that are consistent with many previous analyses. For reference, we generated 20 random models by shuffling the coordinates of the Hi-C map before using the distances as input to the reconstruction program (details in Materials and Methods). Random models were generated in order to test whether the observed patterns in genomic organization are due to particular features in the *S*. *cerevisiae* Hi-C map, and not due to the nature of random fluctuations of polymers, to clusters of genes on the same chromosome or to possible biases in the reconstruction method. In addition, we tested whether signals observed in sparse models were significantly stronger than expected from random models. We observed that even the sparsest map—based on 2,751 interactions—was able to reproduce some important hallmarks of the *S*. *cerevisiae* genomic organization.

All reconstructions were formulated as a non-linear optimization problem (see Materials and Methods), aiming to minimize an objective function that is comprised of the sum of square errors between pairwise distances in the reconstructed model and the set of distances provided as input (interactions). The optimization objective score (the square error between reconstruction and input distances) of the resultant models increases, naturally, with the number of interactions (S2A–S2B Fig), as the number of components being summed increases. We note, however, that when we normalized the objective score by the objective of random models generated from the same number of interactions, the score decreased with the number of interactions (Fig 2A). It appears that, according to this relative criterion, additional data facilitates the convergence of the optimization to a better solution. It is possible that the normalized objective score cannot converge to zero with the number of constraints due to the population based nature of Hi-C data, which isn’t consistent with a *single* reconstructed model, as well as due to other biases in the experiment. Random models attained a significantly higher normalized objective score per interaction than either of the model types.

It is important to mention that the reconstructed solutions for a given input are not deterministic and unique. First, the input set of distances was sampled uniformly and independently 20 times from the total map of distances for each type of model (0.5%-50% sparseness). Second, the optimization process was initialized randomly 4 times for each interaction set / sample and the best solution (out of 4) in terms of optimization error was selected. We studied the similarity of the models generated in this manner (Fig 2B). To this end, Spearman's correlation between all pairwise distances in each model was computed to measure the similarity between different reconstructions with the same degree of sparseness. As expected, the similarity increases with the number of interactions. We note that even when including all interactions in the model, different solutions attained a median correlation of 0.77, a result which is possibly related to the population of genomic conformations that Hi-C measures, as well as to the stochastic nature of the optimization. In comparison, random models with the same (but permuted) interactions attained an average correlation of 0.43. Thus, our randomization scheme was able to generate a wide distribution of models, which lead to large variance in results (Fig 2). The positive correlation attained for random models is due to the fact that some constraints apply also in this case. For instance, genes that are located nearby on a chromosome are expected to remain in proximity in random 3D reconstructions. As a consequence, random models also serve as control for the linear organization of genes on the chromosomes. This provides an explanation for some of the observed results in random models (discussed below).

Centromeres [8] were significantly co-localized in all models, with a normalized set distance (NSD) below 1, and significantly lower than random models (Fig 2C). The strength of co-localization increased with the number of interactions. It is interesting to note that centromeres in random models also exhibited some degree of co-localization. Telomeres were not co-localized in any of the models, although Duan *et al*., based on a different modeling approach, identified inter-chromosomal contact enrichment between telomeres [8]. It is possible that signals detected using one approach will be hard to detect using a different approach. This result may be due to telomere co-localization in multiple, spread clusters (S3 Fig). We did find, however, that telomeres were distributed closer to the nuclear envelope, as indicated by their distance from the center of the nucleus (Fig 2D). An analysis of chromosome arms interactions confirmed that the two arms of each chromosome interact more often than with other arms, as previously observed [8] (Fig 2E); that each arm is closely packed (S2C Fig); and that shorter arms tend to interact more often (S2D Fig). These results suggest that chromosome territories are maintained in the 3D reconstructions.

We observed that early-firing replication origins (Clb5-independent) [27] were co-localized in all models, including random ones (Fig 2F). It had been suggested before, that random conformations of the yeast genome exhibit co-localization of early-firing replication origins [11], in agreement with previous analysis of Hi-C data [8,28]. Thus it is not surprising to find the set co-localized in random models. Co-localization in random models may also be attributed in part to a tendency of early-firing origins to be positioned closer linearly on the chromosome. Specifically, it should be noted that models containing 50% of the interactions (and above) showed significant co-localization of early-firing replication origins even with respect to the co-localized random distribution. We also found that late-firing replication origins were co-localized in all models and more significantly so than early replication origins, compared with random models (Fig 2G). A relation between subnuclear positioning and replication timing has been observed in yeast and other eukaryotes [29,30], suggesting that replication later in S phase takes place at specific loci, such as the nuclear periphery, the nucleolar periphery, and at internal blocks of heterochromatin [31]. Evolutionary chromosome breakpoints were found to be significantly co-localized in models containing at least 5% of the Hi-C interactions (Fig 2H), as suggested by Duan *et al*. [8]. We controlled for an overlapping signal with the co-localization of centromeres by excluding breakpoints that were located in the vicinity of a centromere.

We found the previously identified 2 tRNA clusters [8] co-localized significantly in models containing 5% of the interactions and above (Fig 2I). Genes bound by the same transcription factor (TF) in the vicinity of their transcription start site (TSS) [32] were also significantly co-localized (Fig 2J), as previously observed in [14,33]. Specifically, sites bound by *Irr1p*, part of the cohesin complex, were co-localized (Fig 2K) as suggested in [33]. Repeating this analysis for binding sites in upstream activating sequences (UAS) and open reading frames (ORF) yielded similar however less significant results (S2E–S2F Fig). We analyzed a slim set of Gene Ontology (GO) terms and found no global co-localization of genes relating to the same term (Fig 2L). A set of GO terms has been previously suggested to be co-localized based on *S*. *cerevisiae* Hi-C data [10], and additional terms have been suggested based on *S*. *pombe* Hi-C data [9], but we found no significant support for this conjecture in the 3D reconstructions (S2G Fig). Restating the question, we employed a measure of GO terminology distance between genes that has been shown to correlate with Hi-C-based 3D genomic distances [16], and found it to be correlated with pairwise gene distances in the 3D reconstructions for all models (S2H Fig).

Previous results in *S*. *cerevisiae* [10,14,16] and other eukaryotes [6,9,18] have shown a relation between gene expression and 3D genomic organization. We found the correlation coefficient of co-expression between pairs of genes to be negatively correlated with gene distances, suggesting that co-expressed genes are co-localized (Fig 2M) as suggested by [10,18]; however, this correlation was not significant compared with random models. We tested the correlation between the average expression levels of pairs of genes with pairwise gene distances on the 3D model (S2I Fig) and observed that protein abundance levels were negatively correlated with distance, suggesting a co-localization of highly expressed genes and vice versa. In addition, we considered the distance between genes on a protein-protein interaction (PPI) graph and showed that it is strongly correlated with pairwise distances on the models (Fig 2N), consistent with previous predictions [16] and observations in human [34]. Finally, we verified that the codon usage frequency similarity (CUFS; details in Materials and Methods) of genes—a measure of the functional distance between them [16]—is strongly correlated with 3D distances in the reconstructions (Fig 2O), consistent with previous observations [16]. The latter result enabled us to use predicted distances from CUFS as means to improve 3D reconstructions as described in the following sections.

**Fig 3:**
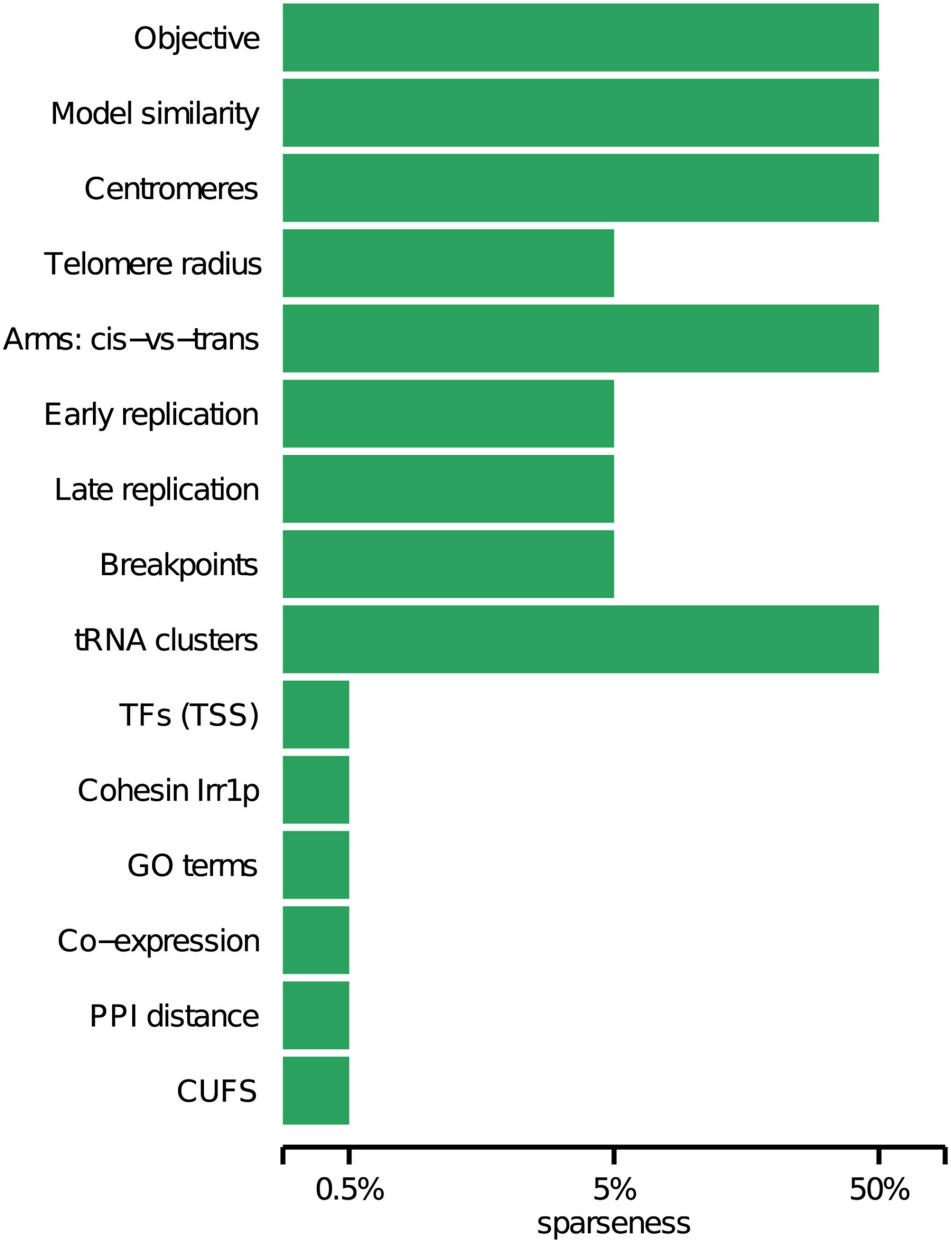
Minimal reproduction of the complete dataset. The bars in the figure denote the minimal amount of data required to reproduce the results obtained using the complete dataset in Fig 2. The minimal degree of sparseness is the one that for all models with equal or larger datasets we were unable to reject the null hypothesis that the median of values was equal to that of the complete dataset (Wilcoxon two-tail rank sum at 0.01 significance level). In most cases (10 out of 15), 5% of the data sufficed to reproduce the result observed for 100%. Choosing a significance threshold of 0.05 affected only model similarity and *Irr1p* (minimal data: 100% and 50%, respectively).

We estimated the minimal amount of data that is required to reproduce the results obtained by models generated from the complete dataset. We considered a sparse result to be similar to the full result if we were unable to reject the null hypothesis that the two medians of the value distributions were equal (Wilcoxon two-tail rank sum at 0.01 significance level). For example, if the co-localization of TFs was similar for all models with sparseness of 0.5% and above, we concluded that the minimal sparseness required to reproduce this result was 0.5% (see Fig 3). We repeated the process for each of the tests in Fig 2. It can be seen that in most cases (10 out of 15) as little as 5% of the data could reliably reproduce results from models based on the complete data. In none of the tests the complete data was required.

These results may indicate that the scale of the Hi-C experiment could be reduced if 3D reconstruction is employed to analyze the data. We attempted to test this hypothesis by repeating the sparse reconstruction process with a modified approach, aiming to simulate a smaller experiment. In this scheme, rather than randomly sampling from the complete map of distance constraints, we gradually damped the number of observed reads (frequency) between each pair of DNA fragments in the experiment. We then proceeded with the reconstruction, regarding the input Hi-C map as a complete new dataset (details in Materials and Methods). Surprisingly, a 20-fold damped Hi-C map showed significantly stronger signals on most of the tests (S4 Fig). This could be explained by the fact that contacts that were retained after a 20-fold damp were significantly enriched in the original dataset and are probably related to distinct architectural features of yeast genomic organization.

### *S*. *cerevisiae* reconstructions can be improved by integrating *S*. *pombe* data

One advantage of 3D reconstructions over statistical analysis of raw Hi-C measurements is that additional data can be incorporated into their construction and taken into account in subsequent analyses. Reconstructed models can thus be improved by integrating genomic datasets of different types. One way by which this can be achieved, is by adding constraints to the optimization problem, as proposed by [8]. Alternatively, we propose the addition of components to the objective function of the optimization problem. These additional components should contain 3D interactions that are non-overlapping with the Hi-C interactions at hand, thus only filling gaps in our knowledge for unknown genomic loci (Fig 1; details in Materials and Methods). We also employed a heuristic for distributing the additional information by sampling with higher probability from regions with low coverage in the original Hi-C map (details in Materials and Methods). Specifically, we propose that integrating 3D genomic measurements from different organisms may improve the reconstructed models. Our approach is motivated by previous results that suggested that 3D genomic organization of orthologous genes tends to be conserved [15,16]. In addition, data integration may facilitate overcoming biases that are organism-specific and protocol-specific.

**Fig 4:**
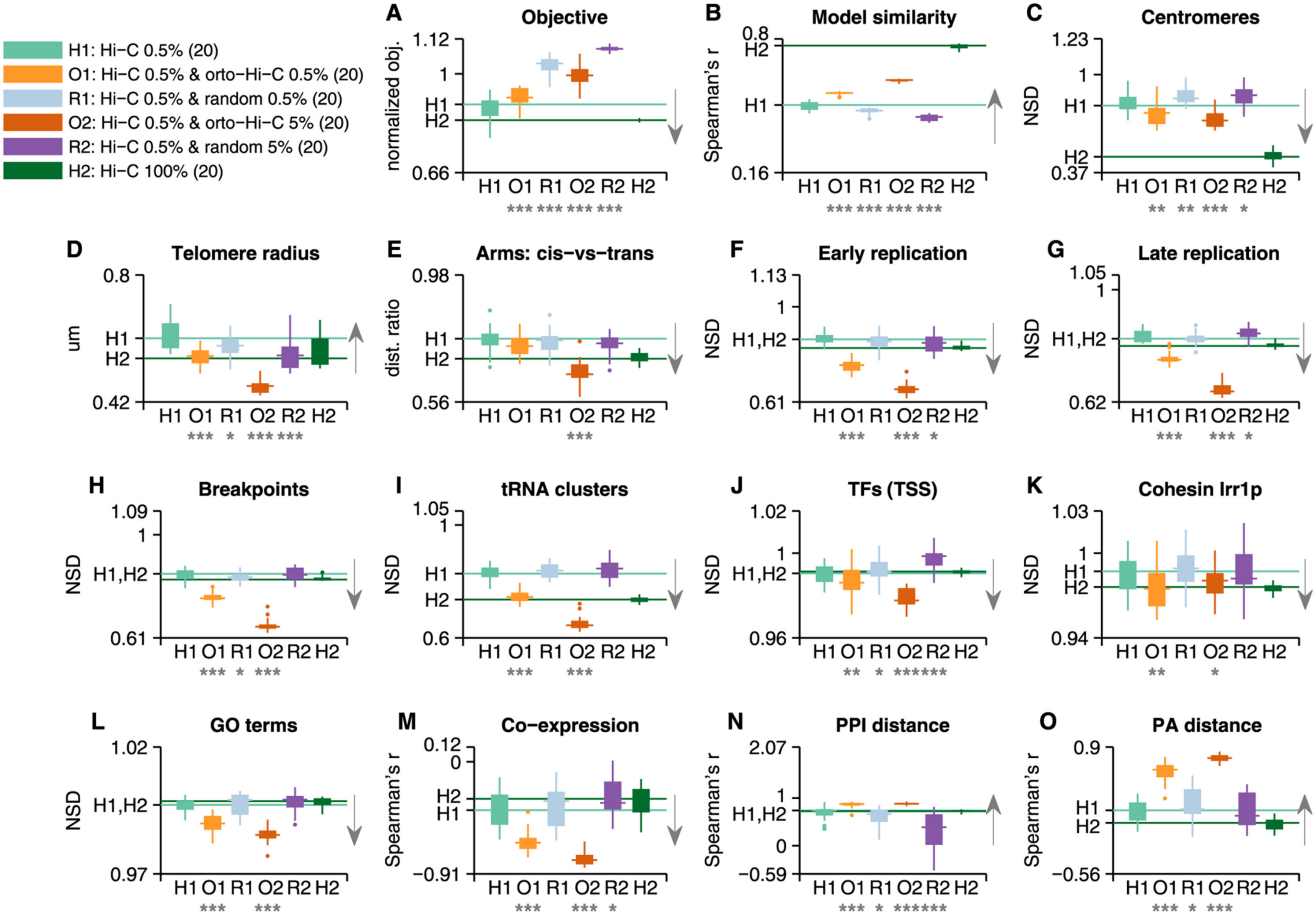
Orthologous-integrated 3D reconstructions. The figure contains benchmark test results for 2 types of *S*. *cerevisiae* 3D genomic models incorporating additional *S*. *pombe* Hi-C interactions (*orto-Hi-C*) and 2 types of models incorporating additional random interactions (*random*). 20 models were generated from each type. Random models were generated by permuting the coordinates of the original *S*. *cerevisiae* Hi-C map (see Materials and Methods). Results in each panel compare the improved reconstructions with the baseline model—*Hi-C-0*.*5%*. Arrows denote the expected direction for an improved model (a stronger signal than the one appearing in Fig 2). We observe that in most tests the addition of orthologous interactions shows a significant improvement over the baseline model (marked by the horizontal line H1) and over models containing additional random interactions. Moreover, some models show stronger signals than 100% models (marked by the horizontal line H2). Results that are distributed significantly above or below the baseline according to Wilcoxon signed rank (one-tail), are denoted with a star or more with respect to their significance level (one star for p<0.05, two for p<0.01, three for p<0.001). **(A)** Optimization objective function of the reconstructed solution, normalized with respect to random models with similar properties. **(B)** Average Spearman’s correlation between the pairwise distances in each model (9.1x10^5^ points) with the other reconstructions generated in its category. **(C)** Centromere co-localization, measured in normalized set distance (NSD), expected to be lower/greater than 1 for co-localized/dispersed sets, respectively. **(D)** Telomere radius from the center of the nucleus. **(E)** Ratio of the average *cis* (intra-chromosomal) distances between chromosome arms and *trans* (inter-chromosomal) distances. **(F)-(L)** Co-localization results for various sets of functional loci. Where the set comprises of several co-localized subsets (such as each GO term, tRNA clusters 1 and 2, etc.), the result presented is the mean of the sets’ mean distance. **(M)** Spearman's correlation between pairwise distances of genes and their coefficient of correlation of expression (*n* = 2,000 bins). **(N)** Spearman's correlation between pairwise distances of genes and their distances on a protein-protein interaction (PPI) graph (*n* = 2,000 bins). **(O)** Spearman's correlation between pairwise distances of genes and their protein abundance (PA) distances—measuring the similarity in expression levels (*n* = 2,000 bins).

We generated 20 models based on 0.5% of the Hi-C map with the addition of an equal number of orthologous Hi-C measurements, and 20 models with 10-fold the number of interactions (5%) transformed from *S*. *pombe* Hi-C maps (Fig 1B). In addition, 20 random models were generated with the addition of 0.5% and 5% of the interactions from permuted *S*. *cerevisiae* Hi-C maps (Fig 1D). We then analyzed the reconstructions similarly to the previous section in order to determine whether the additional interactions contribute to the reconstructed models and improve upon them (Fig 4). By performing a comparison with integrated random models, we tested whether the improvement seen on these quality tests is only due to the increase in the number constraints and whether the cumulative improvement on the tests is likely to be observed by chance. On most tests, orthologous-integrated models (*orto-Hi-C*) scored higher than the baseline 0.5% models: The addition of 2,751 (*orto-Hi-C* 0.5%) interactions significantly improved (Wilcoxon one-tail signed rank at 0.01 significance level) on 0.5% Hi-C models in 12 out of 15 quality tests, compared with random models that significantly improved the reconstructions in none of the tests (permutation test for *orto-Hi-C* 0.5% *vs*. random: p<10^–4^, details in Materials and Methods). The addition of 27,506 interactions (*orto-Hi-C* 5%) significantly improved on 0.5%-HiC in 12 quality tests while random interactions improved in none of them (*orto-Hi-C* 5% *vs*. random p<10^–4^). We note that the latter *orto-Hi-C* models performed even better on 10 quality tests than models comprising of the complete dataset.

We normalized the objective score of *orto-HiC* models by the objective of random models generated from the same number of interactions, sampled from shuffled Hi-C maps and shuffled orthologous-Hi-C maps. We observed that the normalized objective function value is smaller than random (<1) for *orto-Hi-C* models, but increases with the number of added orthologous interactions (Fig 4A). Thus, it appears to be harder for the process to converge to models that are compatible with numerous orthologous interactions. It is also clear that the similarity between models was improved for *orto-Hi-C* (Fig 4B).

Centromeres were significantly more co-localized in integrated models (Fig 4C; p_0.5%_ = 1.5x10^-3^, p_5%_<10^–4^). However, there was no improvement in the signal for telomeres, which moved away from the nuclear periphery towards the center of the nucleus (Fig 4D). This issue could be avoided by constraining the telomeres to the nuclear periphery in the reconstruction program [9,11]. Chromosome arms analysis showed an increase in intra-chromosomal interactions (Fig 4E, p_5%_<10^–3^).

The most apparent improvement was found in functional model features, such as early- and late- firing replication origins (Fig 4F and 4G), evolutionary breakpoints (Fig 4H) and tRNA clusters (Fig 4I; Wilcoxon one-tail signed rank p<10^–4^ for each test). Improved results were seen also for all TFs around the TSS (Fig 4J; p_0.5%_ = 3x10^-3^, p_5%_ = 5.6x10^-5^) and particularly Cohesin *Irr1p* (Fig 4K; p_*0*.5%_ = 0.008, p_5%_ = 0.0297), as well as correlation with PPI distance (Fig 4N, p<10^–3^). Moreover, results that were expected, but not significant, in Hi-C reconstructions, improved considerably, such as GO terms co-localization (Fig 4L; p<10^–4^), correlation with the co-expression coefficient of genes (Fig 4M; p<10^–4^) and protein abundance (PA) distance between genes (Fig 4O; p<10^–4^). We note that, in general, the signal increased with the addition of more orthologous-interactions to the models in the aforementioned tests.

We repeated the above process while integrating orthologous-interactions into other baseline models. 5% sparse Hi-C models (S5 Fig) showed similar results (sig. improvement on 12 tests). The FDR-corrected Hi-C map employed by Duan *et al*. [8] (S6 Fig) also showed comparable results (sig. improvement on 10 tests), indicating that the integrated orthologous interactions are not redundant with the highly significant reads in this dataset. We also repeated the integration with distance constraints obtained from a 200-fold damped Hi-C map, as described above and in Materials and Methods (S7 Fig), leading to a significant improvement on 11 tests. Finally, we tested whether orthologous-interactions can contribute to a reconstructed model based on the complete Hi-C map (S8 Fig). The coverage of gene-related data (orthologous-interactions) and the vast genomic areas already covered by the complete Hi-C dataset limited the number of additional interactions to 20% of the data. As a result, the weight of the additional interactions in the reconstruction problem is smaller than it was when integrated into sparse Hi-C maps. Nevertheless, orthologous-integrated models led to improvement in 9 of the quality tests (7 of which were significant), including a decrease in the normalized objective of the reconstruction. Thus, this demonstrates that orthologous-interactions contain additional information that was missing from the Hi-C dataset and resulted in a better convergence of the reconstruction.

### Reconstructions can be improved by integrating predicted functional interactions between genes

In this section, we considered additional forms of genomic data which can provide heuristics for the reconstruction of better, biologically meaningful 3D models. To this end, we employed a previously proposed tool that can serve as a proxy for functional similarity between genes, the codon usage frequency similarity (CUFS) [16]. Given a pair of genes, the CUFS metric gives an estimate of the functional distance between them (see Materials and Methods); this distance was shown to be highly correlated with the 3D genomic organization of eukaryotic genes, including *S*. *cerevisiae* (see also Fig 2O), as well as with many functional features [16]. These results provide motivation for the addition of CUFS-interactions to the optimization problem in order to improve 3D reconstructions. The main advantage of CUFS over other existing annotations is that it is computed based solely on the sequence of ORFs in the genome, and thus it is expected to be less biased than most of the currently available genome-wide interactions datasets, and can provide complete coverage for any given sequenced genome.

**Fig 5:**
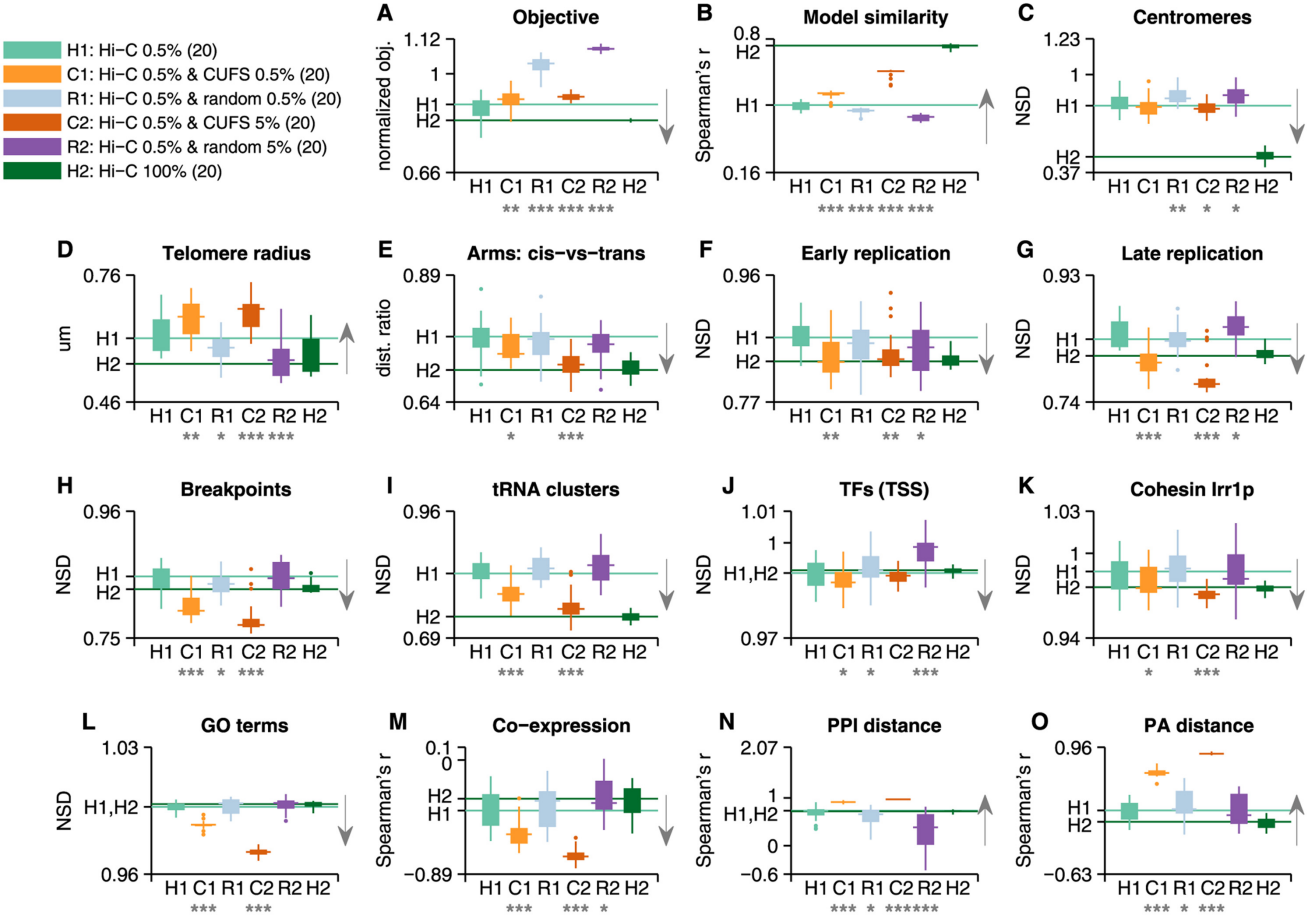
CUFS-integrated 3D reconstructions. The figure contains benchmark test results for 2 types of *S*. *cerevisiae* 3D genomic models incorporating additional interactions based on the codon usage frequency similarity between genes (*CUFS*) and 2 types of models incorporating additional random interactions (*random*). 20 models were generated from each type. Random models were generated by permuting the coordinates of the original *S*. *cerevisiae* Hi-C map (see Materials and Methods). Results in each panel compare the improved reconstructions with the baseline model—*Hi-C-0*.*5%* (marked by the horizontal line H1). Moreover, some models show stronger signals than 100% models (marked by the horizontal line H2). Arrows denote the expected direction for an improved model (a stronger signal than the one appearing in Fig 2). We observe that in most tests the addition of CUFS interactions shows a significant improvement over the baseline model and over models with random interactions. Results that are distributed significantly above or below the baseline according to Wilcoxon signed rank (one-tail), are denoted with a star or more with respect to their significance level (one star for p<0.05, two for p<0.01, three for p<0.001). **(A)** Optimization objective function of the reconstructed solution, normalized with respect to random models with similar properties. **(B)** Average Spearman’s correlation between the pairwise distances in each model (9.1x10^5^ points) with the other reconstructions generated in its category. **(C)** Centromere co-localization, measured in normalized set distance (NSD), expected to be lower/greater than 1 for co-localized/dispersed sets, respectively. **(D)** Telomere radius from the center of the nucleus. **(E)** Ratio of the average *cis* (intra-chromosomal) distances between chromosome arms and *trans* (inter-chromosomal) distances. **(F)-(L)** Co-localization results for various sets of functional loci. Where the set comprises of several co-localized subsets (such as each GO term, tRNA clusters 1 and 2, etc.), the result presented is the mean of the sets’ mean distance. **(M)** Spearman's correlation between pairwise distances of genes and their coefficient of correlation of expression (*n* = 2,000 bins). **(N)** Spearman's correlation between pairwise distances of genes and their distances on a protein-protein interaction (PPI) graph (*n* = 2,000 bins). **(O)** Spearman's correlation between pairwise distances of genes and their protein abundance (PA) distances—measuring the similarity in expression levels (*n* = 2,000 bins).

We normalized CUFS distances according to the *S*. *cerevisiae* Hi-C distance map, transforming them to estimated nanometric distances (Fig 1C; details in Materials and Methods) and added the resultant interactions to the reconstruction objective function. We then tested the models to see whether the additional interactions improved upon the Hi-C-0.5% model (Fig 5). We generated 20 models based on HiC-0.5% maps, containing additional 2,751 (*CUFS 0*.*5%*) interactions and observed that the addition improved results compared to the baseline model in 14 out of 15 quality tests (10 of which were significant, Wilcoxon one-tail signed rank at 0.01 significance level) while the corresponding random models significantly improved on the baseline results in none of the tests (permutation test for *CUFS-0*.*5% vs*. random: p<10^–4^, details in Materials and Methods). The addition of 27,506 (*CUFS-5%*) interactions significantly improved the results in 12 quality tests while the corresponding random models significantly improved in none of them (*CUFS-5% vs*. random p<10^–4^). We note that *CUFS-5%* showed stronger signals than the ones observed in *Hi-C 100%* on 9 quality tests, despite being based on considerably sparser data.

We normalized the objective score of CUFS models by the objective of random models generated from the same number of interactions, sampled from shuffled Hi-C maps and shuffled CUFS maps. We observed, similarly to orthologous-integrated models, that the normalized objective is lower than random (<1), but still higher than the baseline (Fig 5A). Model similarity significantly improved for CUFS models (Fig 5B; p<10^–3^). Centromeres were marginally more co-localized in CUFS models compared to the baseline Hi-C models (Fig 5C; p_5%_ = 0.044), while telomeres occupied regions closer to the nuclear envelope (Fig 5D; p<0.01). Chromosome territories were more compact in CUFS models (Fig 5E, p_0.5%_ = 0.02, p_5%_<10^–3^). Many functional sets were significantly more co-localized in CUFS-integrated models compared with the baseline Hi-C model, such as early- (Fig 5F; p<0.01) and late-firing replication origins (Fig 5G; p<10^–3^), evolutionary breakpoints (Fig 5H; p<10^–3^), tRNA clusters (Fig 5I; p<10^–3^). Global TFs co-localization (Fig 5J) was improved for 0.5% models (p_0.5%_ = 0.0175) but not for 5% models, however *Irr1p* bound genes (Fig 5K; p_0.5%_ = 0.048, p_5%_ = 5.4x10^-4^) were significantly more co-localized than before. Other functionally related features that improved include GO terms (Fig 5L; p<10^–4^), as well as the correlation between pairwise gene distances and co-expression coefficient (Fig 5M; p<10^–3^), PPI distances (Fig 5N; p<10^–4^) and similarity in expression levels (Fig 5O; p<10^–4^). We repeated the above process while integrating CUFS-interactions into other baseline models, as was done for orthologous-interactions, with comparable results (S5–S8 Figs). Markedly, CUFS-interactions were able to improve models based on the complete Hi-C dataset in 13 out of 15 quality tests (12 of which were significant).

## Discussion

In this study, we proposed an approach for the reconstruction of 3D genomic models. Our conclusions are that at least according the current quality tests the redundancy in Hi-C measurements in *S*. *cerevisiae* is great; thus, if we aimed at studying 3D reconstructions we could do it with significantly sparser amount of data. We were able to reproduce many of the previously reported results on yeast genomic organization for the first time in 3D reconstructions. We also suggest that distances on a protein-protein interactions graph are correlated with gene pairwise distances on the genomic models. In addition, 3D reconstructions can be improved using our proposed approach to generate models that reproduce previous results better. We applied the proposed method to several baseline models and demonstrated significant improvement on a set of quality tests. The ability to improve models which were based on the complete Hi-C dataset suggests that our predicted distances may contain valuable information that is complementary to the *S*. *cerevisiae* Hi-C dataset. Our results support previous observations that the genomic organization of genes is partially conserved between species [16]. These results also support observations that codon usage is tightly linked to functional relatedness of genes and to spatial genomic organization [16]. The proposed approach is not limited to a particular reconstruction method, and can be easily employed in different reconstruction schemes and in other organisms. The methods proposed here are gene-centered, but can be extended to any type of genomic data. Additional types of data may be employed to improve reconstruction in the future, *e*.*g*., gene expression levels, protein-protein interactions as well as metabolic networks. Improvement of 3D reconstructions can be further extended to integrate several Hi-C datasets from the same organism, where available. In multicellular organisms, tissue-specific data may be employed to improve the reconstruction, such as a list of known active / inactive genes in a cell type, condition, or developmental stage. Hi-C data from different tissues can also potentially be utilized and integrated into the model; however, in this case attention should be given to choosing *tissue-invariant* features, such as some aspects related to the local chromosome folding of topologically associated domains (TADs) [35,36].

There are a number of computational challenges related to 3D reconstruction in higher eukaryotes. First, whole-genome reconstructions have been limited to small genomes due to computational costs [21]. It is possible that in some cases removing some of the constraints can improve the computational time without changing the quality of the result. Second, diploid cells introduce a complication since two copies (often separated spatially [37]) of each chromosome are measured simultaneously in Hi-C but later modeled as two polymers.

Finally, most Hi-C experiments to date were carried on cell populations, thus measuring the average contact frequencies of a *population* of 3D structures. The latter issue can be partially dealt with by producing a population of reconstructions [11,15,18], as was carried here. In the future, single-cell Hi-C [15] will enable to estimate the variability of genomic conformations of different cells and to compare it with the reconstructed model distribution; it would be interesting to explore the performance of the approach described here on single-cell Hi-C.

## Materials and Methods

### Sequences

Sequences of the *S*. *cerevisiae* (S288c) and *S*. *pombe* (972h) genomes were obtained from NCBI, as well as the coding sequences of their 5,888 (SC) and 5,123 (SP) protein-coding genes.

### Hi-C data preparation

Hi-C data was obtained from [8]. Hi-C map (HindIII library) values were corrected using the iterative correction proposed by [38]. We utilized the complete (unfiltered) set of contacts for model reconstruction, allowing for the optimization program to reconstruct the most probable chromosome conformation given the entire dataset. The conversion from Hi-C contacts to nanometric distances was performed as proposed in [8], by generating a profile of linear genomic distances (in bp) vs. Hi-C contact frequency using 100 bins. We employed linear interpolation between bins, free of any assumptions on the contact frequency distribution. Conversion from genomic distances to nanometric distances was then approximated by a 130 bp/nm constant describing characteristic chromatin packing [8]. The frequency-to-distance function was applied on the Hi-C contact map after binning it according to the reconstruction model coordinates (10kbp resolution)—summing the contacts in each bin (S9 Fig). In S6 Fig, the 1% FDR-corrected Hi-C map was obtained from [8], corrected using iterative correction and its distance conversion function was learned as described above.

### 3D model reconstruction

3D reconstructions were generated using a modified version of the program provided by [8]. The program defines a non-linear programming optimization problem and utilizes the Ipopt (version 3.6.1) package to solve it [39]. The reconstruction objective function being minimized is the sum of square errors between the current solution and the given 3D distances:

$$\min\sum_{i}{\left(dist\left(p_{i},q_{i}\right)-\delta_{i}\right)}^{2}$$ (1)

Where *p*_*i*_,*q*_*i*_ are a pair of beads in the model and δ_*i*_ is their expected distance (the input to the program). The solution is bounded, to comply with the following constraints [8]: nucleus radius; maximal elasticity between two adjacent beads; minimal distance between chromosome polymers; position of the nucleolus; position of chromosome XII's centromere. The optimization is initialized with a random configuration of the chromosomes. Models typically converged after approximately 1800 iterations. Models were visualized (S1 Fig) using Jmol [40]. The reconstruction program is available for download at: http://www.cs.tau.ac.il/~tamirtul/reconstruction.zip

### Sparse reconstruction

Sparse models were generated by uniformly sampling a portion of the set of Hi-C interactions (0.5%, 5%, 50%). Hi-C interactions were binned at a resolution of 10kbp prior to sampling. Sampling was repeated independently 20 times (generating 20 sets of interactions), from each set 4 models were constructed (for a total of 80 models). The best model out of the 4 in terms of optimization error (the dual infeasibility score as reported by Ipopt) was chosen to represent the set of sampled interactions, in order to avoid solutions that converged to local minima. For the sake of consistency, the 80 models generated from the 100% map were partitioned into 20 groups and selected according to the same principle.

In S4 and S7 Figs, damped Hi-C maps (simulating a smaller-scale experiment) were generated by: (1) dividing the read count of each pair of HindIII fragments by the damping factor (*e*.*g*., 20); (2) filtering reads below 1 (a detection threshold); (3) quantizing the reads by rounding them to the nearest integer; (4) summing the reads per 10kbp bin; (5) converting the observed contact frequencies to distances according to rank, *i*.*e*. by assigning the highest value in the damped-map with the lowest value in the distribution of Hi-C distances and so on, until the sparse damped-map was completely converted.

### Orthologous Hi-C interactions

Hi-C maps for *S*. *pombe* were obtained from [9]. The authors also provided with an experimentally verified (via FISH) nanometric distance function to convert the Hi-C measurements for this map. Each *S*. *pombe* pair of genes was assigned with a spatial distance according to the nearest measured coordinates on the map. Next, 3,367 orthologous families obtained from the manually curated orthologs database at PomBase [41] were utilized to transform distances from pairs of *S*. *pombe* genes to their identified respective orthologs in *S*. *cerevisiae* (averaged on multiple genes). So that, given a distance matrix **D** ^B^ in organism B, the orthologous-transformed matrix in organism A is given by:

$$D_{ij}^{\text{B}\to \text{A}}=\frac{1}{\left|O_{i}\right|\left|O_{j}\right|}\sum_{k\in O_{i}}\sum_{l\in O_{j}}D_{kl}^{\text{B}}$$ (2)

Where *O*_*j*_ is the set of orthologous genes in organism B corresponding to gene *j* in organism A. 3D distances were further normalized to account for the different dimensions of the nucleus in the 2 organisms, by scaling *S*. *pombe* distances to have the same median as the *S*. *cerevisiae* set of Hi-C distances (S10 Fig). We filtered the resultant distances, taking the bottom 5% (based on more reliable Hi-C contacts) as additional constraints for improving the *S*. *cerevisiae* reconstructions. Using different thresholds up to 50% did not have a strong effect on the results. When integrating orthologous-distances into the 100% Hi-C map (S8 Fig), we raised the threshold to 50% in order to increase the coverage of the orthologous map. Distances were binned into model coordinates (10kbp resolution) by averaging the distances in each bin.

Finally, orthologous interactions were sampled to produce an additional set of interactions added to Hi-C interactions (see main text), after excluding bins with existing Hi-C interactions (see Fig 1B). The sampling scheme chosen (poor-get-richer, see below) aims at distributing the data added to the model while reinforcing parts of the model that were missing / badly represented in the original Hi-C data. To this end, we computed the degree of each bead in the model—*i*.*e*., how many interactions with other beads are currently known for that bead. The probability to add an interaction between a pair was chosen to be proportional to the inverse of the beads’ degrees. This resulted in a distribution of degrees with smaller variance.

Poor-get-richer sampling:

**Data:** distance matrix *C*.

**Result:** distance matrix *C* containing additional *n* interactions.

**Init:** compute the degree of each node according to:

$$d_{i}=\max\left(0.5,N\left\{C_{i,j}>0,\forall j\right\}\right)$$

where *N*{} indicates the number of elements satisfying the condition. A non-zero degree was assigned for beads with no interactions, for numerical stability.

**begin**

1. Remove orthologous interactions *(i*,*j)* if distance already known (*C*_*i*,*j*_ > 0)**for** *each sample up to n*
2. Draw from the available orthologous interactions *(i*,*j)* with probability:
  $$p_{i,j}\propto d_{i}^{-1}d_{j}^{-1}$$
3. Update node degrees.

### CUFS interactions

The codon usage frequency similarity (CUFS) [16] was used as a proxy for functional distances between genes. Codon frequencies were computed for each of the 5,888 *S*. *cerevisiae* ORFs. The CUFS distance between a pair of genes, given their two codon frequency vectors, **p** and **q** (normalized for a sum of 1), was then computed by the Endres-Schindelin metric for probability distributions [42]:

$$d_{KL}\left(p,q\right)=\sum_{i=1}^{64}p_{i}\log\left(\frac{p_{i}}{q_{i}}\right)$$ (3)

$$m\equiv \frac{1}{2}\left(p+q\right)$$ (4)

$$d_{ES}=\sqrt{d_{KL}\left(p,m\right)+d_{KL}\left(q,m\right)}$$ (5)

Where *d*_KL_ is the Kullback—Leibler divergence. In order to transform CUFS distances to nanometric distances independently of additional data, we used a simple linear model. CUFS distances were converted to nanometric distances by scaling the map to have the same median as the Hi-C distances. The top and bottom 10% of distances were considered for integration into the Hi-C distance map (S11 Fig). Different thresholds did not have a strong effect on the result.

The resultant distance map was binned into model coordinates (10kbp resolution) by averaging the distances in each bin. CUFS interactions were sampled to produce an additional set of interactions added to Hi-C interactions (see Fig 1C), after excluding bins with existing Hi-C interactions. To this end, the poor-get-richer sampling scheme was employed.

### Random interactions

Random interactions were obtained by permuting the coordinates of the 10kbp-binned Hi-C interaction map. Thus, the permuted maps preserve the distribution of distances in the dataset. 20 permutations were drawn, and the complete permuted maps were utilized to generate the random reconstructions that appear in Fig 2. The success rate for the convergence of the reconstruction program was about 75%. Since we kept the best model out of 4 (in terms of Ipopt’s optimization error) for every model reported in the study, this low failure-rate did not pose a problem. The squared reconstruction error between the input distance matrix and reconstructed model was significantly higher for random models than for non-permuted Hi-C models (real: 7.6x10^4^ μm^2^, random: 8.9x10^4^ μm^2^). The magnitude of this error is related to the number of interactions in the input matrix, which was identical for random and real. When testing for co-localization of sets of elements in random models, we used their original position on the chromosome (rather than their position on the permuted coordinates).

In addition, random sets were sampled and added to a subsampled, non-permuted Hi-C distance map using poor-get-richer sampling (see Fig 1D), after excluding bins with existing Hi-C interactions as described above. The resultant perturbed models appear in Figs 4 and 5.

### Co-localization

Co-localization / dispersion analysis of sets of loci / genes was performed using a normalized set distance (NSD) measure. NSD is defined as the mean set distance for the set of interest, normalized by the expected distance of random sets of equal size drawn from the same model. Specifically, 100 samples were drawn from all possible gene pairs, for gene sets; or samples from a uniform 10kbp coordinate grid on the model, for other sets of loci (such as early replication origins). Thus, the obtained value is <1 for co-localized sets and >1 for dispersed sets.

### Chromosome arms analysis

Chromosome arms analysis followed that of [8] and provides quantified analysis of chromosome territories and the interactions between chromosomes. To this end, three measures were employed: first, the ratio of the average distance between short arms (<250kbp, as defined by Duan *et al*.) and long arms (>250kbp) (*short-vs-long*); second, the ratio of the average distance between the two arms of each chromosome and the rest of the arms (*cis-vs-trans*); third, the ratio of the average distance between regions on the same arm and their distance from other arms (*self-vs-else*). All distances along the arms were sampled in 10kbp resolution.

### Correlation

Model similarity quantifies how similar repeated reconstructions are according to pairwise distances. Model reconstruction is stochastic due to both random initialization of the reconstruction program, as well as random sampling of interaction sets. Model similarity was estimated by computing the correlation between pairwise distances in 10kbp resolution between all generated models in the same category. For example, a total of 80 models were generated in the Hi-C-0.5% category (4 instances from 20 sampled sets of interactions). For each of the 80x80 possible pairs of models the pairwise distances between 10kbp-spaced genomic loci were utilized to compute Spearman’s rank correlation between them. Similarity was defined as the average correlation coefficient between all pairs.

Spearman’s rank correlation with CUFS, protein abundance [43,44] distance (normalized distance between pairs of PA: 2|*p-q*|/(*p+q*), see Figs 4 and 5), average protein abundance of pairs (see S2 Fig), Gene Ontology (GO) [45,46] term distance [16], protein-protein interaction graph distance and gene co-expression was computed by dividing all gene pairs into 2,000 bins.

### P-values

P-values, unless stated otherwise, were computed using a one-tailed Wilcoxon signed rank test, comparing the distributions of values obtained for 2 model types (*e*.*g*., HiC-0.5% vs. an orthologous-integrated model). We chose a paired-test in order to compare each set of sampled Hi-C interactions to its extended set (the one containing additional interactions).

In order to estimate the probability of observing the resultant improvement on tests in integrated models by chance, we shuffled the models between two categories—*e*.*g*., random-integrated models and orthologous-integrated models—10,000 times. We then computed the total benchmark result for the drawn partition. If the probability of observing the difference between models (or a more extreme one) was smaller than 0.01 we rejected the null hypothesis that the observed improvement on the tests was a random effect.

### Additional datasets

Centromere positions were obtained from [8]. Telomeres were defined as the first and last beads in each chromosome. 77 early-firing replication origins and 123 late-firing replication origins were obtained from [27]. 718 evolutionary breakpoints were obtained from [47] (WolfeScerKwalBreakpoints.txt), and 127 breakpoints within 50kbp from centromeres were excluded from this set. Two tRNA clusters were obtained from [8], where they were suggested to be co-localized in the nucleus. TF gene binding data was obtained from [32], and included 193 TF binding profiles around transcription start sites (TSS), 194 TF binding profiles in upstream activating sequences (UAS) and 159 TF binding profiles inside the ORF. Cohesin *Irr1p* co-localization was computed by averaging the result over the 3 libraries. Co-expression correlation coefficients were computed between the mRNA expression profiles of genes in 530 conditions [46]. Full GO annotations were obtained from SGD [46] and mapped onto the generic GO slim definitions. The set of previously co-localized GO terms [10] appearing in S2 Fig included: GO:0016798, GO:0016810, GO:0016874, GO:0006810, GO:0006950, GO:0007049, GO:0051276, GO:0007165, GO:0007059, GO:0005694, GO:0005886, GO:0005730. Protein-protein physical interactions were obtained from several databases [48–51] and filtered according to quality score thresholds 200 and 0.3 for STRING and the rest, respectively. When computing shortest path distances on the PPI graph, disconnected pairs were assigned with a finite large number (255).

### Clustering

We performed hierarchical clustering (see S3 Fig) by utilizing MATLAB’s linkage function on the pairwise telomere distance matrix (averaged across 80 models), using the average distance algorithm.

## Supporting Information

S1 Fig Examples of reconstructed models. Models are not illustrated in precise, equal scale. **(A)-(D)** Reconstructed models for different levels of sparseness. **(E)** A random model. **(F)-(G)** Integrated models, containing predicted distances in addition to the complete Hi-C dataset. **(H)** An illustration of the proposed reconstruction approach employed in (F)-(G).
(EPS)

S2 Fig Additional analysis of sparse 3D reconstructions. **(A)** Optimization objective function (squared error between input distances and resultant model). **(B)** Mean objective per input constraint. **(C)** Ratio of the average distance between points on a chromosome arm and the average distance between that arm and the rest of the chromosome arms. **(D)** Ratio of the average distance between short chromosome arms (<250kbp) and long chromosome arms (>250kbp). **(E)** Co-localization results for TF binding sites in the upstream activation sequence (UAS) library. **(F)** Co-localization results for TF binding sites in the open reading frame (ORF) library. **(G)** Co-localization results of previously identified GO terms (details in Materials and Methods). **(H)** Spearman's correlation between pairwise distances of genes and their Gene Ontology terminology distance (n = 2,000 bins; details in Materials and Methods). **(I)** Spearman's correlation between pairwise distances of genes and their average protein abundance (PA; n = 2,000 bins).
(EPS)

S3 Fig Telomere clusters. The figure shows the pairwise distance heat map between telomeres, averaged across 80 reconstructions, and ordered by utilizing hierarchical clustering on the Hi-C 100% model. Telomeres from the long chromosome arm are denoted with L while ones from the short arm are denoted with S. Additional matrices corresponding to Hi-C 5% (retained signal) and random models (missing signal) are presented for reference. The largest cluster (1) comprises of 15 telomeres from 11 chromosomes (upper corner, marked with an asterisk). Additional clusters appear along the diagonal. In addition, it can be seen that the 2 telomeres of each chromosome tend to interact. Distances are in micrometers.
(EPS)

S4 Fig Sparse reconstruction from a damped Hi-C map. The results in this figure mirror those appearing in Fig 2, but reconstructions were generated by damping the contact frequencies in the entire Hi-C map 2-, 20- and 200-fold before proceeding with the reconstruction (details in Materials and Methods).
(EPS)

S5 Fig Integrated reconstruction based on 5% sparse Hi-C maps. The results in this figure mirror those appearing in Figs 4 and 5, with the difference that predicted distances were integrated into 5% sparse Hi-C maps, with an equal number of predicted and Hi-C distances. Orthologous-integrated models improved on 12 out of 15 tests (12 of them were significant), while CUFS-integrated models improved on 13 (13 of them were significant).
(EPS)

S6 Fig Integrated reconstruction based on FDR-corrected Hi-C maps. The results in this figure mirror those appearing in Figs 4 and 5, with the difference that predicted distances were integrated into FDR-corrected Hi-C maps, with an equal number of predicted and Hi-C distances. P-values here were computed using Wilcoxon rank-sum test, since the FDR corrected map was fixed in all 20 reconstructions (unlike sparse reconstructions, where maps were resampled and paired with their corresponding integrated models). Orthologous-integrated models improved on 10 out of 15 tests (10 of them were significant), while CUFS-integrated models improved on 10 (10 of them were significant).
(EPS)

S7 Fig Integrated reconstruction based on damped Hi-C maps. The results in this figure mirror those appearing in Figs 4 and 5, with the difference that predicted distances were integrated into 200-fold damped Hi-C maps, with an equal number of predicted and Hi-C distances. P-values here were computed using Wilcoxon rank-sum test, since the damped map was fixed in all 20 reconstructions (unlike sparse reconstructions, where maps were resampled and paired with their corresponding integrated models). Orthologous-integrated models improved on 11 out of 15 tests (11 of them were significant), while CUFS-integrated models improved on 12 (11 of them were significant).
(EPS)

S8 Fig Integrated reconstruction based on the complete Hi-C data. The results in this figure mirror those appearing in Figs 4 and 5, with the difference that predicted distances were integrated into the 100% Hi-C map, with predicted interactions amounting to one fifth of the constraints in the original map. P-values here were computed using Wilcoxon rank-sum test, since the 100% map was fixed in all 20 reconstructions (unlike sparse reconstructions, where maps were resampled and paired with their corresponding integrated models). CUFS-integrated models improved on 13 (12 of them were significant), while orthologous-integrated models improved on 9 out of 15 tests (7 of them were significant).
(EPS)

S9 Fig Distribution of Hi-C distances. This figure presents the histogram of distances in the Hi-C distance map (100 bins).
(EPS)

S10 Fig Distribution of orto-HiC distances. This figure presents the histogram of the normalized *S*. *pombe* distance map (100 bins), after transformation to *S*. *cerevisiae* coordinates (orthologous genes) and scaling. For the purpose of reconstruction, distances up to the marked threshold were sampled. These distances are expected to be more reliable. Note that the median is equal to the one in S9 Fig.
(EPS)

S11 Fig Distribution of CUFS distances. This figure presents the histogram of the scaled CUFS distance map (100 bins). For the purpose of reconstruction, distances below the left marked threshold and above the right threshold were sampled. Note that the median is equal to the one in S10 and S9 Figs.
(EPS)
